# Multicentric evaluation of high and low power lasers on RIRS success using propensity score analysis

**DOI:** 10.1007/s00240-024-01535-w

**Published:** 2024-02-10

**Authors:** Eren Erol, Gokhan Ecer, Murat Can Kiremit, Mehmet İlker Gokce, Mehmet Balasar, Ahmet Furkan Sarikaya, Muammer Babayigit, Umut Can Karaarslan, Elif Ipek Aksoy, Kemal Sarica, Kamran Ahmed, Selçuk Güven

**Affiliations:** 1 Department of Urology, Meram School of Medicine, Necmettin Erbakan University, Konya, Turkey; 2 Department of Urology, Konya State Hospital, Konya, Turkey; 3 Department of Urology, School of Medicine, Koc University, Istanbul, Turkey; 4 Department of Urology, Ankara University School of Medicine, Ankara, Turkey; 5 Department of Urology, Sancaktepe Sehit Prof. Dr. Ilhan Varank Research and Training Hospital, Istanbul, Turkey; 6 Khalifa University, Abu Dhabi, United Arab Emirates; 7 MRC Centre for Transplantation, King’s College London, London, UK

**Keywords:** Flexible ureterorenoscopy, Kidney stone, Retrograde intrarenal surgery, High-power laser, Low-power laser

## Abstract

In this study, we aimed to evaluate the effect of HPL on different parameters by different centers and urologists. While doing this, we evaluated different parameters by comparing HPL(High Power laser) and LPL(Low-power laser). This is an observational, retrospective, comparative, multicentric study of prospectively organised database. A total of 217 patients who underwent RIRS for kidney stones smaller than 2 cm in three different centers were included in the study. The patients were divided into two groups; LPL used (Group1, n:121 patients) and HPL used (Group2, n:96). Propensity score matching was done in the data analysis part. After matching, a total of 192 patients, 96 patients in both groups, were evaluated. There was no difference between the groups regarding age, gender, stone side, and stone location. The stone-free rate on the first day was 80.3% in Group 1, it was 78.1% in Group 2 (*p* = 0.9). In the third month, it was 90.7% in Group 1 and 87.5% in Group 2 (p:0.7).Hospitalization duration was significantly higher in Group 1. (2.35 ± 2.27 days vs. 1.42 ± 1.10 days; *p* < 0.001).The operation duration was 88.70 ± 29.72 min in Group1 and 66.17 ± 41.02 min in Group2 (*p* < 0.001). The fluoroscopy time (FT) was 90.73 ± 4.79 s in Group 1 and 50.78 ± 5.64 s in Group 2 (*p* < 0.001). Complications according to Clavien Classification, were similar between the groups(*p* > 0.05). According to our study similar SFR and complication rates were found with HPL and LPL. In addition, patients who used HPL had lower operation time, hospital stay, and fluoroscopy time than the LPL group. Although high-power lasers are expensive in terms of cost, they affect many parameters and strengthen the hand of urologists thanks to the wide energy and frequency range they offer.

## Introduction

Percutaneous nephrolithotomy (PNL), retrograde intrarenal surgery (RIRS), and extracorporeal shock wave lithotripsy (ESWL) are considered optimal options for kidney stone treatment [1]. For kidney stones smaller than 2 cm, flexible ureteroscopy (FURS) with or without shock wave lithoripsy (SWL) remain recommended options by the European Association of Urology (EAU) and the American Association of Urology (AUA) guidelines [2].

The holmium:yttrium–aluminum-garnet (Ho: YAG) laser has been considered the gold standard for laser lithotripsy in the last two decades. Today, RIRS has mainly been used with Ho: YAG laser lithotripsy. [3] Besides being used successfully in lithotripsy, prostate enucleation or ablation, endopyelotomy, tumor ablation, and bladder neck incision are the areas where Ho: YAG is used effectively in urology practice.

Low-power Ho: YAG devices (LPL); lasers with a maximum power of up to 30–35 Watts with a maximum laser pulse frequency are considered. High-power Ho: YAG devices (HPL) identify lasers with power over 35 Watts [3]. HPL features higher pulse energy and higher pulse frequency. It also has the feature of obtaining faster lithotripsy and smaller stone fragments [4]. Although its use has become widespread recently, studies on its efficacy and safety are still limited and ongoing. Our current knowledge is promising that HPL offers more effective treatment in lithotripsy with a shorter operative time.

The literature suggests that HPL may be a better option than LPL; In this study, we aimed to evaluate the effect of HPL on different parameters by evaluating multiple parameters as well as evaluating the use of HPL by different centers and urologists.

## Methods

The local ethics committee approved the study (Necmettin Erbakan University ethics committee 167/2023).

### Type of study

**Fig. 1:**
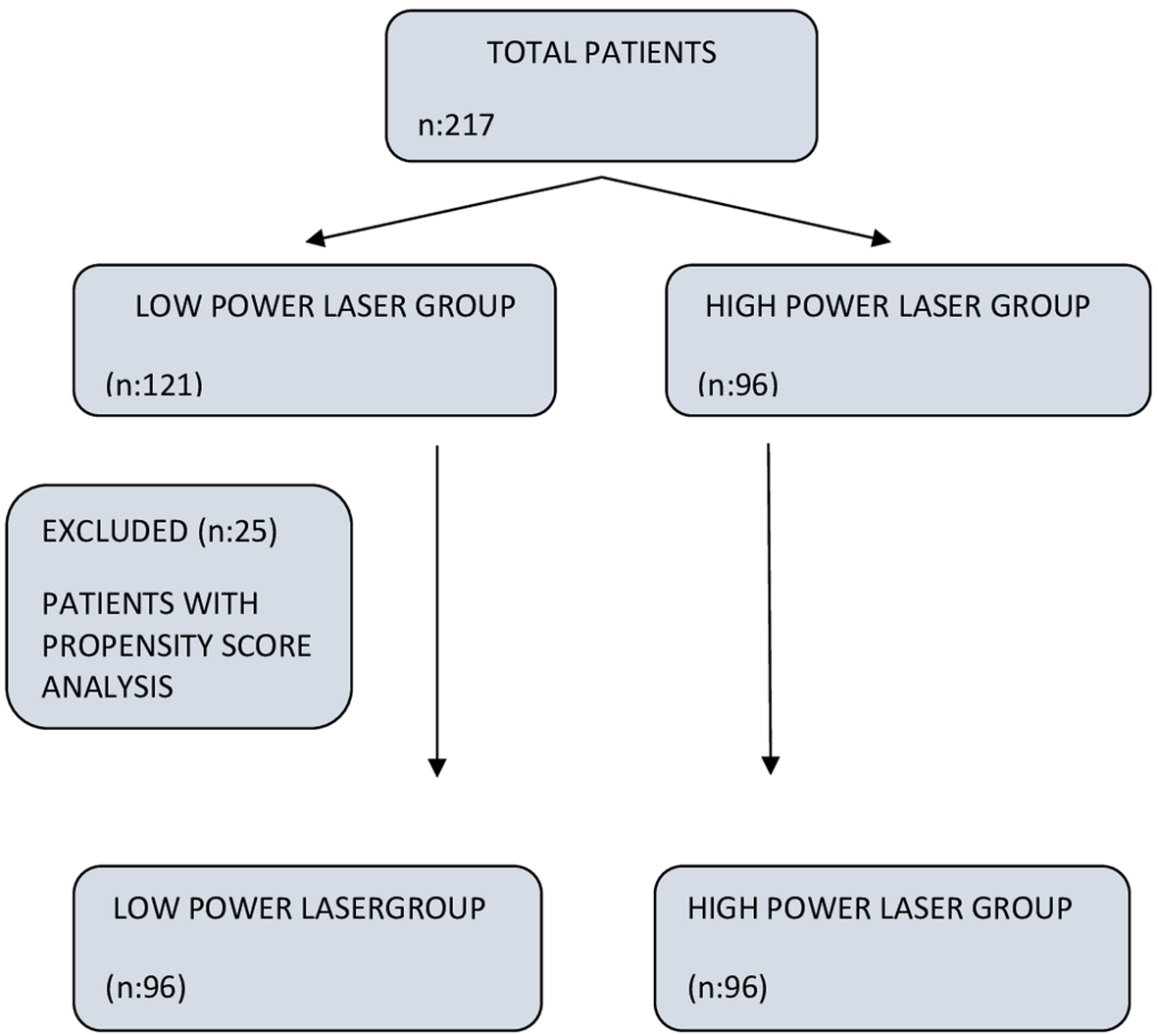
Flowchart of patient selection

This is an observational, retrospective, comparative, multicentric study of prospectively organised database. A total of 217 patients who underwent RIRS for kidney stones smaller than 2 cm between 01 June 2021 and 1 September 2022 in 3 different centers were included in the study. The patients were divided into two groups; LPL used (Group1, n:121 patients) and HPL used (Group2, n:96). Propensity score matching was done in the data analysis part. After matching, a total of 192 patients, 96 patients in both groups, were evaluated (Fig. 1).

An informed consent form was obtained from all patients before the study. Stone characteristics, duration of surgery, Ho: YAG laser energy settings, postoperative complications, and stone-free status in preoperative non-contrast computed tomography (CT) imaging were recorded.

### Inclusion exclusion criteria

Patients between the ages of 18–70 years, with kidney stones smaller than 2 cm and who underwent RIRS for kidney stones were included in the study. Patients younger than 18 years of age and older than 70 years of age, with anatomical anomalies, who had undergone ESWL before, who did not have a preoperative CT scan, whose urine culture was positive, and who did not come for follow-up at the fourth postoperative week were excluded from the study.

### Devices and settings

Flex-Xc, Flex-X2s (Karl Storz, Germany) and HU-32 (Huge-med, China) flexible ureterorenoscope were used at the first Center; Flex-X2S (Karl Storz, Germany) and LithoVue (Boston Scientific, USA) in the second center; Flex-Xc, Flex-X2s (Karl Storz, Germany), Wiscope (OTU Medical) and LithoVue(Boston Scientific, USA)in the third center.

For LPL: 272 μ fiber with lasers, Dornier 30 W (Germany) in the first and second center; and 200 μ fiber with Lumenis VersaPuls P20H (Yokneam, Israel) was used in the third center. 272 μ fiber was used with HPL in the first and second center with Jenna Surgical 150 W (Germany); and 200 μ fiber with Quanta Litho Cyber Ho 150 W in the third center (Samarate, Italy).

### Surgical tecnique and process

All surgical procedures were performed by experienced endourologists. The procedure was performed in the lithotomy position under general anesthesia. A safety guide wire was placed in the kidney under fluoroscopic guidance. All patients underwent ureterorenoscopy with a rigid ureterorenoscope. A second guidewire was placed during ureterorenoscopy.

In patients who used UAS, UAS was advanced over the second guide wire. In patients who did not use UAS, flexible URS was advanced directly to the kidney over the second guidewire. After reaching the stone, the stone was fragmented with the Holmium:YAG laser.

UAS was used according to the surgeon’s preference. The procedure was performed without sheath in patients whose UAS could not be placed. If the procedure could not be performed without UAS, a DJ stent was placed and tried again 1 month later. The procedure was performed after treatment in patients with a positive preoperative urine culture.

According to each departmental regulation, the Double J stent was removed 2–4 weeks after surgery. SFR was defined as the absence of fragments of any size in X-ray and USG performed at four weeks postoperatively.

### Outcome measure

The primary outcome measure was stone-free rate determined by standard care imaging at three months. Intraoperative and postoperative complications, operative time, hospitalization time, and postoperative analgesia were secondary outcome measures. Amount of the energy Joule and frequency used for fragmentation during the procedure, operation time from the start to the end of the procedure in minutes, hospitalization time from the end of the procedure to the discharge in days, and fluoroscopy time (FT) from the beginning to the end of the procedure is stated in minutes as secondary outcome measures.

### Statistical analysis

Statistical analysis was performed with SPSS 25.0 (Statistical Package). Categorical variables are described by frequencies and percentages. Continuous variables are presented as mean and standard deviations. Independent T, Kruskal–Wallis and Chi-square (*χ*^2^) tests were used to compare the relationship between categorical and continuous variables subgroups. A P value below 0.05 was considered statistically significant. Propensity Score matching was used to homogenize the groups in the study.

## Results

Ninety six patients were determined in both groups, and then the analysis was made. There was no difference between the groups regarding age, gender, stone side, and stone location (Table 1). In Group 1, 21 (21.8%) patients had lower pole stones, while 20 (20.8%) had multiple calyceal stones. In Group 2, 19 (19.8%) patients had lower pole stones, while 24 (25.1%) patients had stones in multiple calyces (*p* = 0.118).

**Table 1:** Demographic data

	Group 1 (low-power energy) N:96	Group 2 (high-power energy) N:96	*p* value
Age (years ± SD)	46,8 ± 17,8	51,3 ± 19,4	0,08
Weight (kg ± SD)	69 ± 13,5	73,7 ± 18,3	0,07
Height (cm ± SD)	165,5 ± 16,4	165,9 ± 15,3	0,85
BMI (kg/m^2^ ± SD)	24 ± 3,8	25,7 ± 4,5	0,08
PreoperativesCr (mg/dl ± SD)	0.98 ± 0.38	1.09 ± 0.58	0.6
PostoperativesCr (md/dl ± SD)	0.95 ± 0.32	1.02 ± 0.37	0.3
PreoperativeEgfr	82,2 ± 19,7	82,7 ± 32	0,88
PostoperativeEgfr	86,2 ± 18,9	83 ± 28	0,328
PreoperativeHb (g/dl)	13,2 ± 1,8	13,2 ± 2	0,97
PostoperativeHb (g/dl)	12,7 ± 1,8	12,7 ± 2	0,81
Gender			
Male	63	55	0,144
Female	33	41	
High-power lasertype			
HoYAG	96(%100)	96(%100)	1
Surgical experience			
< 50 cases	0	0	1
> 50 cases	96(%100)	96(%100)	
Anti tromboticuse			
No	91(%95)	85(%88,5)	0,077
Yes	5(%5)	11(%11,5)	
Preoperative urine culture			
Negative	82(%85,4)	83(%86,5)	
Positive	14(%14,5)	13(%13,5)	0,914
Previousstonesurgery			
No	64(%66,6)	58(%60,4)	0,912
Yes	32(%33,4)	38(%39,6)	
ASA Score			
1	34(%35,4)	38(%39,6)	
2	51(%53,1)	47(%49)	0,578
3	11(%11,5)	10(%10,4)	
4	0	1(%1)	
Type of isolated bacteria			
E Coli	5(%5)	11(%11,4)	
Enterokok	3(%3,3)	2(%2,1)	0,276
Candida	1(%1)	3(%3,1)	
Acinetobacter	4(%4,1)	2(%2,1)	
Klebsiella	1(%1)	1(%1)	
Type of prophylactic antibiotic			
Ceftriaxon	84(%87,5)	90(%93,6)	
Ertapenem	10(%10,4)	3(%3,2)	0,256
Meropenem	0	1(%1,1)	
Flukonazol	2(%2,1)	2(%2,1)	
Stone size (mm ± SD)	11,6 ± 4,6	12 ± 2,9	0,513
Stone density(HU ± SD)	1150 ± 366	1146 ± 265	0,925
Stone number (number ± SD)	1,74 ± 1,2	1,9 ± 1,4	0,345
Stone side			
Right	48(%50)	47(%49)	0,927
Left	48(%50)	49(%51)	
Stone location			
Lower calyx	21(%21,8)	19(%19,8)	
Middle calyx	13(%13,6)	11(%11,5)	0,118
Upper calyx	8(%8,3)	9(%9,4)	
Pelvis	34(%35,5)	33(%15,2)	
Multiple	20(%20,8)	24(%25,1)	
Stone impaction			
No	54(%56,2)	56(%58,3)	0,943
Yes	42(%43,8)	40(%41,7)	
Preoperative hydronephrosis			
No	60(%62,5)	60(%62,5)	
Yes	36(%37,5)	36(%37,5)	0,863
Preoperative stenting			
No	75(%78,1)	81(%84,4)	0,342
Yes	21(%21,9)	15(%15,6)	
Urinary tractanomaly			
No	90(%93,7)	86(%89,6)	
Yes	6(%6,3)	10(%10,4)	0,207
Comorbidities			
No	73(%76)	71(%74)	0,523
Yes	23(%24)	25(%26)	

While dusting, fragmentation, and popcorn techniques were used less alone in Group 1, it was observed that the combined technique was preferred more than Group 2 (*p* < 0.001). When the combined fragmentation technique was compared, it was used in 82 (85.4%) patients in Group 1 and 39 (39.6%) patients in Group 2. Stone fragmentation was observed in 2 (2.1%) patients in both groups.In the examination of the laser settings used; the mean frequency value used was 10.3 in Group 1, the mean was 37.9 in Group 2; the average energy level used was 1.8 J in Group 1; It was observed that the mean was 0.5 J in Group2 (*p* < 0.001).

*Stone-free rate*: While the stone-free rate on the first day was 80.3% in Group 1, it was 78.1% in Group 2 (*p* = 0.9). In the third month, it was 90.7% in Group 1 and 87.5% in Group 2; there was no significant difference between the groups (*p*:0.7). The preoperative serum creatinine value was 0.98 ± 0.38 mg/dl in Group 1 and 1.09 ± 0.58 mg/dl in Group 2, the postoperative serum creatinine value was 0.95 ± 0.32 mg/dl in Group 1 and 1.02 ± 0.37 mg/dl in Group 2, and there was no difference between the groups (respectively; *p* = 0.6; *p* = 0.3) (Table 2).

**Table 2:** Post-operative data

	Group 1(low-power energy) N:96	Group 2(high-power energy) N:96	*p* value
Fragmentation method			
Dusting	2(%2,1)	34(%35,4)	
Fragmentation	10(%10,4)	21(%21,9)	
Popcorn	2(%2,1)	2(%2,1)	** < 0,001**
Combined	82(%85,4)	39(%39,6)	
Ureteral access sheath use			
No	37(%38,5)	36(%37,5)	0,745
Yes	59(%61,4)	60(%62,5)	
Auxilliary procedure			
No	55(%57,2)	67(%69,8)	
URS	13(%13,6)	11(%11,5)	
RIRS	13(%13,6)	12(%12,5)	0,13
ESWL	10(%10,4)	5(%5,2)	
Other	5(%5,2)	1(%1)	
Post-operative stent use			
No	8(%8,3)	3(%3,1)	
Yes	88(%91,7)	93(%96,9)	0,076
Complication			
Clavien1	12(%12,5)	18(%18,7)	
Clavien 2	4(%4,1)	4(%4,2)	0,15
Clavien 3	0	0	
Clavien 4	0	0	
Stone-free status PO first day			
No	19(%19,7)	21(%21,9)	
Yes	77(%80,3)	75(%78,1)	0,9
Stone-free status PO first-third month			
No	9(%9,3)	12(%12,5)	
Yes	87(%90,7)	84(%87,5)	0,7
Preferred pulse energy (J ± SD)	1,8 ± 0,4	0,5 ± 0,35	** < 0,001**
Preferred pulse requency(Hz ± SD)	10,3 ± 1,8	37,9 ± 17,9	** < 0,001**
Prefered pulse width(ms ± SD)	393 ± 96	1700	** < 0,001**
Operation time(min ± SD)	88.70 ± 29.72	66.17 ± 41.02	** < 0,001**
Floroscopy time(sn ± SD)	90.73 ± 4.79	50.78 ± 5.64	** < 0,001**
Hospital stay(day ± SD)	2.35 ± 2.27	1.42 ± 1.10	** < 0,001**
Cost per case(Tl ± SD)	1562 ± 576	3152 ± 1346	** < 0,001**

Significant findings are shown in bold

*Hospitalization time*: Hospitalization duration was significantly higher in Group 1. (2.35 ± 2.27 days vs. 1.42 ± 1.10 days; *p* < 0.001).

*Operation time*: The operation duration was 88.70 ± 29.72 min in Group1 and 66.17 ± 41.02 min in Group2 (*p* < 0.001). The FT was 90.73 ± 4.79 s in Group 1 and 50.78 ± 5.64 s in Group 2 (*p* < 0.001) (Table 2). The correlation analysis found a positive correlation between the operation duration, fragmentation time, FT, and hospitalization (*p* < 0.001) (Table 2).

*Complications*: Auxiliary procedure requirements, complications according to Clavien Classification, UAS usage, and postoperative stent placement were similar between the groups(*p* > 0.05) (Table 2).

## Discussion

The lasers’ power in treating urinary tract stones ranges from 10 to 140 watts (W). In research and clinical practice, lasers > 35 W are accepted as HPL and those with < 35 W as LPL [3, 4]. While low-power holmium lasers can be used for lithotripsy and some other urological interventions except prostate resection, they offer advantages with lower purchasing costs. HPL, on the other hand, is claimed to provide faster disintegration and minimize the need for baskets when removing stones during lithotripsy. Apart from stones, it is also used in various reconstructive surgical procedures with endoscopic prostate intervention.

Our study found similar SFR and complication rates with HPL and LPL. In addition, patients who used HPL had lower operation time, hospital stay, and FT than the LPL group. Previous studies comparing HPL and LPL reported similar SFR at both laser powers [5–7]. Furthermore, SFR was similar for the first and postoperative third months at different energies.

Pietropaolo et al. reported operation durations of 52.02 ± 27.90 min in patients who used LPL; and 38.46 ± 22.88 min in patients using HPL (*p* < 0.001)[5]. Another study by Shrestha et al. reported 38 (19–60) min in the LPL group and 40 (25–60) min in the HPL group [6]. Moreover, Golomb et al. reported the mean operation duration as 53 (15–168) min [8]. However, stone sizes and measurement techniques vary widely between studies. In our study, the operation time in the LPL group was 88.7 ± 29.5 min and 66.1 ± 41 min in the HPL group. We think the longer surgery duration in our study may be due to the larger stone diameter and higher cumulative mean stone length.

The relationship between FURS complications, operative times, and the thermal effect of the laser on the tissue has been reported previously [9, 10]. Complications increase by prolonged operation time and increased intrarenal pressure [10, 11]. Our study reports postoperative complications according to the Clavien-Dindo grading [11, 12]. In the literature comparing HPL and LPL as complications, the complication rates were changing between 4.3–21% vs. 4.7–17.7% for HPL and LPL, respectively (5,6,8); it was 22.9–18.2% for HPL and LPL in our study, and no difference was found in terms of complications between the groups. Although serum creatinine measurement is nonspecific, it indirectly indicates tubular damage, and preoperative and postoperative creatinine values and kidney damage markers were considered [13, 14]. In the study of Ertas et al., the preoperative serum creatinine value was 0.97 ± 0.58 mg/dl, while the post-op serum creatinine value was 1.0 ± 0.61 mg/dl [14]. A study found that the serum creatinine level, which was 0.89 ± 0.22 preoperatively, increased to 0.98 ± 0.25 in the postoperative measurement in patients who underwent RIRS using UAS [13, 14]. We did not determine a significant difference between the HPL and LPL groups; however, further studies with more specific kidney damage markers are needed to clarify the effect of different energy sources and power settings on kidney functions.

Sfoungaristos et al., evaluating fluoroscopy times on patients who underwent URS and RIRS, reported that the mean fluoroscopy times were 41.4 s for a more experienced surgeon and 91 s and 44.9 s for two junior surgeons. The procedure time, postoperative double-J stent use, and less surgical experience were independent predictors of increased FT [15]. In our study, the duration of FT in the LPL group was higher than in the HPL group, which can be due to the increased operation time in the LPL group.

### Limitations of the study

The retrospective design and the fact that the interventions were performed in different centers and by different surgeons is a significant limitation of our study. Therefore, propensity matching was done to homogenize the groups. Also, a significant limitation was using different lasers in different settings in both LPL and HPL. The absence of a standardized lithotripsy setting resulted in heterogeneity between groups. Similarly, measuring blood biochemistry in different centers may make a difference when evaluating the data. This study was designed retrospectively, and homogenization between groups was provided by propensity score analysis. In this study, we showed the effects of different power lasers on the success of RIRS. However, more prospectively designed, multicentric studies needed to be conducted in selected patient groups to determine the optimal energy sources and settings.

## Conclusion

It is seen that HPL and LPL are used with similar success and complication rates, and the duration of operation, hospitalization, and FT is reduced with HPL. Studies using properly designed and appropriate biomarkers will better understand how it affects kidney functions in the medium and long term. Although high-power lasers are expensive in terms of cost, they affect many parameters and strengthen the hand of urologists thanks to the wide energy and frequency range they offer.
